# DLC-ac4C: A Prediction Model for N4-acetylcytidine Sites in Human mRNA Based on DenseNet and Bidirectional LSTM Methods

**DOI:** 10.2174/0113892029270191231013111911

**Published:** 2023-11-22

**Authors:** Jianhua Jia, Xiaojing Cao, Zhangying Wei

**Affiliations:** 1 School of Information Engineering, Jingdezhen Ceramic University, Jingdezhen, 333403, China

**Keywords:** N4-acetylcytidine site prediction, DenseNet, Bi-LSTM, channel attention mechanism, deep learning, ensemble learning

## Abstract

**Introduction:**

N4 acetylcytidine (ac4C) is a highly conserved nucleoside modification that is essential for the regulation of immune functions in organisms. Currently, the identification of ac4C is primarily achieved using biological methods, which can be time-consuming and labor-intensive. In contrast, accurate identification of ac4C by computational methods has become a more effective method for classification and prediction.

**Aim:**

To the best of our knowledge, although there are several computational methods for ac4C locus prediction, the performance of the models they constructed is poor, and the network structure they used is relatively simple and suffers from the disadvantage of network degradation. This study aims to improve these limitations by proposing a predictive model based on integrated deep learning to better help identify ac4C sites.

**Methods:**

In this study, we propose a new integrated deep learning prediction framework, DLC-ac4C. First, we encode RNA sequences based on three feature encoding schemes, namely C2 encoding, nucleotide chemical property (NCP) encoding, and nucleotide density (ND) encoding. Second, one-dimensional convolutional layers and densely connected convolutional networks (DenseNet) are used to learn local features, and bi-directional long short-term memory networks (Bi-LSTM) are used to learn global features. Third, a channel attention mechanism is introduced to determine the importance of sequence characteristics. Finally, a homomorphic integration strategy is used to limit the generalization error of the model, which further improves the performance of the model.

**Results:**

The DLC-ac4C model performed well in terms of sensitivity (Sn), specificity (Sp), accuracy (Acc), Mathews correlation coefficient (MCC), and area under the curve (AUC) for the independent test data with 86.23%, 79.71%, 82.97%, 66.08%, and 90.42%, respectively, which was significantly better than the prediction accuracy of the existing methods.

**Conclusion:**

Our model not only combines DenseNet and Bi-LSTM, but also uses the channel attention mechanism to better capture hidden information features from a sequence perspective, and can identify ac4C sites more effectively.

## 1 INTRODUCTION

Nucleoside modifications in epi transcriptomics are essential cellular processes necessary for organisms to function [1]. More than 170 nucleoside modifications in RNA have been identified, including 7-methylguanosine (m7G), N1-methyladenosine (m1A), N6-methyladenosine (m6A), 5-hydroxymethylcytosine (hm5C), 5-formylcytosine (f5C), and N4-acetylcholine (ac4C) [1]. In eukaryotic and prokaryotic tRNAs, rRNAs, and mRNAs, ac4C is an evolutionarily conserved nucleoside modification. The formation of ac4C is mainly catalyzed by N-acetyltransferase 10 (NAT10) [2-4]. Ac4C enhances the fidelity of protein translation and its stability in tRNA and regulates the heat resistance of organisms [5-7]; in rRNA, ac4C performs a vital function for the precision of protein transformation and biosynthesis [8]; in mRNA, ac4C can enhance the steadiness of mRNA and improve its translation efficiency [9, 10]. In addition, research has proven that ac4C is related to the progression, prognosis, and development of more than a few human diseases, along with inflammation, metabolic diseases, autoimmune diseases, and cancer [11-14].

In recent years, with the advancement of high-throughput sequencing technology, Arango *et al.* [9a] used RNA acetylated RNA immunoprecipitation (acRIP) sequencing to detect more than 4000 ac4C loci in human transcriptome mRNA. In addition, quite a few biophysical or biochemical strategies have been developed for ac4C detection, such as high-performance liquid chromatography-mass spectrometry (MS), borohydride sequencing, high-resolution liquid chromatography (HPLC), and antibody assays [14-16]. Yet, all of them require considerable human resources and materials, particularly for relatively large data sets. Therefore, there is a need to develop calculation methods that can precisely and reliably identify ac4C.

Over the past few years, researchers have developed a variety of machine-learning predictors to identify RNA post-translational modification sites [17-20]. There are fewer machine learning predictors for ac4C sites. For example, Zhao *et al.* [21] developed a predictor called PACES, which uses position-specific dinucleotide sequence profile and k nucleotide frequency for feature encoding and uses two random forest classifiers to identify ac4C sites. Alam *et al.* [22] incorporated six nucleotide encoding methods (one-hot encoding, nucleotide chemical properties, nucleotide density, K-mer, EIIP, and PseEIIP) into their model XG-ac4C, using the extreme gradient boost (XGBoost) algorithm to characterize RNA sequence feature information and predict ac4C sites. Su *et al.* [23] proposed a new method based on gradient enhanced decision tree (GBDT), called iRNA-ac4C. The model is based on three feature extraction methods, including nucleotide composition, nucleotide chemistry, and accumulated nucleotide frequencies, to identify ac4C sites in human mRNA. However, this machine learning-based prediction method is backward, only applicable to small sample data, often requires complex feature encoding methods, and has poor prediction performance.

With the development of deep learning, it has been widely used in the field of bioinformatics [24-26], including protein structure prediction [27-29], tumor origin tissue inference [30, 31], and RNA post-transcriptional modification site identification [32-37] due to its great potential. A few researchers have also applied deep learning to ac4C site prediction. For example, Zhang *et al.* [38] introduced the CNNLSTMac4CPred model, which extracted the semantic features of sequences by using a CNN and an LSTM network, combined the semantic feature information, k-nucleotide frequencies, and pseudo-ternary nucleotide composition as the input encoding of sequences, and finally used XGboost as the classification algorithm. Wang *et al.* [39] constructed a predictor called DeepAc4C based on CNN, which uses a mixture of physicochemical patterns and distributed nucleic acid representations to predict sites. However, they only used a relatively simple neural network model, and it is worth noting that convolutional neural networks suffer from the disadvantage of network degradation. Therefore, we can construct a more accurate prediction model for identifying ac4C sites using a simple encoding method and a more sophisticated deep-learning network.

Although research on RNA ac4C site prediction has been conducted for several years, there are still great challenges in mining the information implicit in RNA sequences, which is the focus of this study. To this end, we propose a new deep learning-based network structure, DLC-ac4C, to identify ac4C modification sites in mRNAs, which mainly consist of DenseNet, Bi-LSTM, and channel attention, where “D” stands for the DenseNet module, “L” stands for the Bi-LSTM module, and “C” stands for the channel attention module. In the DLC-ac4C model, three separate encoding methods are used for ac4C sequences: the C2 encoding method, nucleotide chemical properties (NCP), and nucleotide chemical density (ND). Among them, the combination of NCP and ND is usually used to express the nature and frequency of nucleotides [40, 41], while C2 [42] is a denser encoding method than One-hot. First, the three feature codes are synthesized into a feature matrix, and then the feature matrix is fed into a one-dimensional convolutional neural network (1-D CNN) to capture the low-level features of the sequences, and then into DenseNet to obtain the high-level features of the sequences, followed by the introduction of a Bi-LSTM network to obtain the long-term dependencies among the sequences. We use a channel attention mechanism to obtain information about features that have important contributions to the sequence, and the channel attention mechanism is added after DenseNet and Bi-LSTM, respectively. Finally, a fully connected layer is used to receive these high-level features, and a probability value between 0 and 1 is calculated using the SoftMax function. To improve the DLC-ac4C model proposed in this paper, we also used an isomorphic integration [43, 44] approach, where five probability values were obtained using five identical DLC-ac4C network frames, and they were averaged to obtain the final predicted probability. If the value is greater than 0.5, a modification site of ac4C is identified; Otherwise, it is the opposite. The model DLC-ac4C proposed in this article is shown in Fig. (**1**).

The main contributions are summarized as follows:

1) A new DLC-ac4C network structure based on deep learning is proposed to recognize ac4C sites. This model can extract more advanced feature information and capture sequence information more efficiently and has better robustness to locate ac4C sites more accurately.

2) From the perspectives of complete sequence information, nucleotide intrinsic information, and nucleotide frequency and position, we use C2, NCP, and ND to encode features to minimize missing sequence information and maximize RNA sequence feature retention.

3) In order to reduce the generalization error of the model, we use an isomorphic integration method.

The rest of the paper is organized as follows. In Section 2, we presented the dataset used in this paper and the related methods used in the model. Then, we discussed and validated our proposed model in Section 3. Finally, we will summarized our work in Section 4.

## 2 MATERIALS AND METHODS

In this study, we constructed a deep learning-based approach to identify ac4C modification sites in human genomic mRNA. Firstly, the sequence input is converted into numerical vectors through encoding, and then the DLC-ac4C model is trained based on the training dataset. Finally, existing predictors are compared and the model of this study is evaluated.

### 2.1 Benchmark Dataset

The benchmark data in this study comes from the study by Su *et al.* [23]. For the reliability of the data, they selected the cytidine closest to the ac4C peak as the modification site and centered around these modification sites, took 100 nucleotides on both sides as positive samples. Then, again centered on cytidine, the 201nt sequences were randomly selected as negative samples in the non-peak region. Afterward, redundant sequences with higher than 80% similarity were deleted by the CD-HIT [45] tool. Then, they balanced the data set by picking the same number of sequences at random from negative samples as positive samples. Finally, 2206 positive and 2206 negative samples fashioned the training dataset, and 552 positive and 552 negative samples shaped the independent dataset. The benchmark test data set is listed in Table **1**.

### 2.2 Feature Extraction Methods

We have used three feature extraction methods in this work, namely C2 encoding, NCP encoding, and ND encoding, to identify ac4C modification sites in human mRNA, which are described in detail in this section.

#### 2.2.1 C2 Encoding

C2 encoding [42] is a relatively common sequential model for characterizing sequences, which converts elements in biological sequences one by one into specific values from the perspective of preserving global sequence information. Specifically, C2 encoding converts RNA bases on the nucleotide chain of an RNA molecule to 2-bit binary, *e.g*., adenine (A) is coded as (0,0), cytosine (C) is coded as (1,1), guanine (G) is coded as (1,0), and uracil (U) is coded as (0,1). It should be noted that the advantage of the C2 encoding method over the one-hot encoding [46, 47] method is the ease of storage and computation. In this work, the sequence length of each sample is 201nt, so each sequence is transformed into a 201×2 feature matrix after C2 encoding. The encoding process is shown in Fig. (**2**).

#### 2.2.2 NCP Encoding

Nucleotide chemical property encoding (NCP) [48] is an encoding method that extracts intrinsic information between nucleotides. It is well known that different nucleotides have different chemical properties and also possess different functions. Nucleotides A and G are purines and contain two cyclic structures, whereas nucleotides C and U are pyrimidines and contain one cyclic structure; The functional groups are A and G for the amino group and C and U for the keto group; nucleotides C and G contain strong hydrogen bonds, whereas nucleotides A and U contain weak hydrogen bonds. The NCP encoding classifies the four types of nucleotides into three categories defined by the cyclic structure (purine or pyrimidine), functional groups (amino or keto groups) and hydrogen bonds (strong or weak) between them, and Table **2** shows the details.

Suppose we quantify these chemical properties (*x_i_*, *y_i_*, *z_i_*) using a three-dimensional vector representing a given RNA sequence, where *x_i_*, *y_i_*, and *z_i_* are represented as follows.


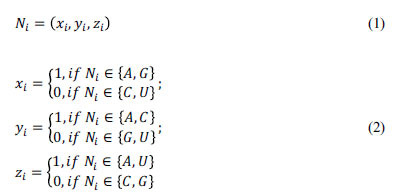


where *x_i_* encodes a nucleotide through a ring structure; *y_i_* encodes a nucleotide through a functional group; and *z_i_* encodes a nucleotide through the strength of a hydrogen bond. As a result, nucleotide “A” can be represented as (1, 1, 1), “U” as (0, 0, 1), “C” as (0, 1, 0), and “G” as (1, 0, 0). In this study, the NCP encoding converts the sequence into a feature matrix of dimension 201×3.

#### 2.2.3 ND Encoding

Nucleotide density (ND) [49] encoding is a frequent approach to encoding in bioinformatics, which represents each RNA sequence by combining information on the nucleotide's frequency and the placement of an individual nucleotide in the sequence. For each RNA sequence, the density *d_i_* of nucleotides *s_i_* at position i is expressed as follows:


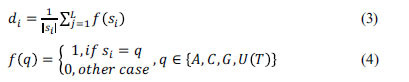


where *L* means the sequence length and 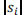 means the length of the 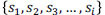 in the sequence. Each RNA sequence can be characterized as a one-dimensional vector after ND encoding. For example, we take a 201nt RNA sample sequence “AGAUCCU…A”. The densities of “A” are 1/1, 2/3, …, 95/201 at positions 1, 3, …, 201; the density of “C” is 1/5 and 2/6 at locations 5 and 6, respectively; the density of “G” is 1/2 at location 2; and the density of “U” is 1/4 and 2/7 at locations 4 and 7, respectively. Thus, ND can encode the sequence as a 201×1 eigenvector. In general, ND encoding is used in conjunction with the NCP encoding method [50, 51]. The encoding process is shown in Fig. (**2**).

### 2.3 Classification Model

In this study, we constructed a deep learning-based model to efficiently capture the deep hidden features of the ac4C locus, called DLC-ac4C. In the DLC-ac4C model, firstly, we transformed the sequence into a 201×6 feature matrix by three feature encoding methods, and subsequently input the feature matrix into a one-dimensional convolutional neural network (1-D CNN) [52] to capture the low-level features of the sequences, then into DenseNet to obtain high-level features of the sequences, followed by the introduction of a Bi-LSTM network [53] to obtain long-term dependencies between the sequences. We use the channel attention mechanism to obtain feature information with important contributions to sequences, and the channel attention mechanism is added after DenseNet and Bi-LSTM, respectively. Thereafter, the obtained feature vectors are fed into a fully connected network, which contains 240 and 40 neurons in the first and second layers, respectively, and the last output layer contains two units for predicting two classes (ac4C samples and nonac4C samples). In addition, SoftMax was chosen as the activation function to calculate a probability value between 0 and 1. Finally, an isomorphic integrated learning approach was used to obtain five probability values using five identical DLC-ac4C network frameworks, and they were averaged to obtain the final predicted probability. The classification results of ac4C loci are determined by the magnitude of the probability values. The DLC-ac4C network framework is shown in Fig. (**1**).

#### 2.3.1 DenseNet

DenseNet [54] is an improvement on the residual network structure (ResNet), building on ResNet a convolutional neural network with dense connections between layers. Unlike the previous direct deepening or widening of network layers, DenseNet establishes a dense connection between adjacent layers, the place every input to the network is a cascade of outputs from all preceding layers, and the feature maps learned by every layer are passed directly to the inputs of all subsequent layers. DenseNet makes full use of sequence features to achieve information flow integration, avoiding the problem of information transfer loss and gradient disappearance between levels, enhancing the transfer between features, obtaining better results with smaller parameters, and extracting advanced features of sequences more effectively. Thanks to the operation of dense connections, early features can additionally be exploited immediately at a deeper level. Fig. (**3**) represents the specific structure of DenseNet.

DenseNet consists frequently of the convolutional layer, the dense block layer, and the transition layer. The low-level feature map of the sequence is initially obtained using one-dimensional convolution, after which multiple dense convolution blocks are concatenated and then down-sampled using a transition layer in order to ensure a uniform size of the feature map, which facilitates the connection between the layers.

The dense block is a structural variant of the CNN that uses dense jump connections to connect every two convolutional layers in the block in a forward propagation manner, allowing for the reuse of low-level features. Its structure is shown in Fig. (**4**). The dense block takes x_0_ as the input and inputs for *x_1_*, *x_2_*, and *x_3_* is an aggregate of all previous layer inputs. The layers within a single dense convolutional block are connected using a nonlinear transformation function that consists of a batch normalization function, a ReLU activation function, and a one-dimensional convolutional layer. In DenseNet, the *L*th level of the model has a total of *L*(*L*+1)/2 connections to the preceding *L*th level, then the output of the L th level is formulated as follows:


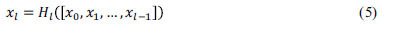


where *H_i_*(.) is the non-linear transformation of layer *L* and 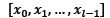 means the splicing operation of the output features from layer 0 to layer *l*-1.

Since the amount of output feature map channels increases after each dense block, in order to limit network parameters and decrease the size of the feature map, we add convolutional and pooling operations between two adjacent dense blocks, called transition layers. The transition layer is composed of a 1×1 one-dimensional convolution and a 2×2 average pooling. The transition layer not only reduces the computational effort but also serves the purpose of feature reduction and compression of the model.

In this work, we repeated the experiments and adjusted the network parameters. The final model used four dense blocks and three transition layers, and the model structure is revealed in Fig. (**1**).

#### 2.3.2 Bi-LSTM

In order to obtain long-term dependencies between sequence features, we used a Bi-LSTM [55, 56] in the model to extract information about the sequence context. The network structure is shown in Fig. (**5**).

The Bi-LSTM consists of two reversed unidirectional LSTM networks that convey information from front to back and back to front respectively, enabling the Bi-LSTM model to integrate forward and backward information of sequences and capture interdependencies between sequences.

The LSTM [57] comprises three gates, an input gate, an oblivion gate, and an output gate. Fig. (**6**) illustrates a schematic diagram of the LSTM cell. Specifically, the characteristic of the forgetting gate is to selectively forget the records stored in the memory unit at the previous moment, and the job of the input and output gates is to control the inputs and

outputs of the memory unit sent to the rest of the network. The LSTM is calculated by the following formula:


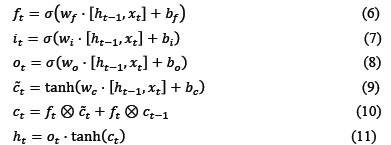


where *i_i_* controls the input to the input gate *c_t_*, *f_t_* controls the memory level *c*_*t*-1_ of the forgetting gate, and o_t_controls the output of tanh (c_t_) W denotes the weight matrix, *b* is the bias vector and 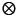 denotes element multiplication. Since the activation function is a sigmoid function, the values of *i_t_*, *f_t_*, and *o_t_* lie between 0 and 1. Furthermore, the Bi-LSTM concatenates the forward and backward hidden states of each base as the output at time step t, with


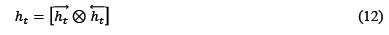


#### 2.3.3 Channel Attention Mechanism

In this study, we introduce a channel attention mechanism [58, 59] to improve the efficiency of model learning, directing the network to focus on feature channels with greater weights, and Fig. (**7**) shows the structural details of the channel attention mechanism. For a feature map with H*W*C, which has several channels C. The value of each channel is first calculated one by one *via* the global average pool and the global maximum pool, then fed to each of the two fully linked layers, and the two outcomes output through the fully linked layer are then summed. This is followed by a Sigmoid activation function that restricts the weights to between 0 and 1 to obtain the weights for each channel. Finally, the extended channel coefficients are multiplied by the initial feature information to give the feature information a new weight, causing the model to draw attention to the more important feature information.

In this study, we wished to seize as many vital features in the sequence as possible. Whereas some of the different channels of the feature map contain more feature information and some contain less. Treating each channel equally would lack the flexibility to treat channels with different weights. In this study, the channel attention mechanism is added to the network model to weigh the target features, making feature extraction more directional to improve the efficiency of sequence feature extraction.

#### 2.3.4 Ensemble Learning

Ensemble learning is a method of fusing individual predictors through voting systems or other strategies that can produce better predictive performance [60]. It is well known that integration across multiple or individual models using appropriate inheritance strategies can enable complementary learning of training data, thereby greatly improving the reliability, accuracy, and efficiency of the model. Therefore, in this study, we used a common integrated learning based on the same model. The difference is that we chose to use five models with the same parameters for the integration operation and used simple averaging as the integration strategy for classification. We refer to this operation as an isomorphic ensemble, and it is effective in reducing the model's generalization error. In this study, we used ten-fold cross-validation by randomly dividing the training dataset into ten equal parts, one of which was taken out each time as the validation dataset, and the other nine parts were employed to train the model. Each of these data sets had the opportunity to be taken as a validation set to measure the models trained on the other nine data sets. Five models were trained and the test set was put into each of the five models to obtain five predictions, and then the final classification results were obtained by calculating the average, where all five models were the same model framework.

#### 2.3.5 Hyper Parameter Setting Instructions

In this section, we introduce the DLC-ac4C network structure and hyperparameters for training. In our experiments, we use NVIDIAGeForceRTX3080TiGPU to train the neural network for the DLC-ac4C model. In model training, we use cross entropy as the loss function, optimize the loss function using Adam optimizer [61], and use gradient descent to adjust parameters to minimize the loss function. Meanwhile, we employ L2 regularization, dropout [62], and early stop [63] to avoid overfitting. In addition, we determined the optimal hyperparameters through comparative experiments. All parameter settings and model training were based on Python 3.8 and Keras 2.8.0 for the DLC-ac4C model. Table **3** shows all hyperparameters for the DLC-ac4C model.

#### 2.3.6 Performance Evaluation

In this study, four commonly used classifier evaluation metrics were selected to evaluate the predictive performance of the DLC-ac4C model, including sensitivity (Sn), specificity (Sp), accuracy (Acc), and Mathews correlation coefficient (MCC). These are defined as:


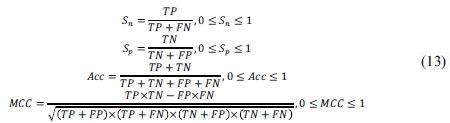


Where TP, FN, TN, and FP indicate the quantities of true positives, false negatives, true negatives and false positives, respectively. Sn and Sp characterize the proportion of ac4C sites and nonac4C sites correctly predicted, respectively. Acc is an indicator of the overall accuracy of the differentiated sample, and MCC is used to precisely evaluate the overall performance of the model.

Furthermore, receiver operating characteristic (ROC) [64] curves were introduced to evaluate the overall performance of the model. The area under the ROC curve (AUC) is calculated and the AUC value is between 0 and 1. The value of AUC is positively correlated with prediction performance, and the greater the value of AUC, the better the overall performance of the model.

Cross-validation is a type of statistical analysis that has been used to check the performance of classifiers and has been widely applied to a variety of classification problems [65-67]. In this study, the robustness of DLC-ac4C was evaluated using tenfold cross-validation, and independent tests were used to compare the performance of DLC-ac4C with existing predictors.

## 3 RESULT AND DISCUSSION

This section begins with a discussion of feature encoding methods and ablation experiments on the network structure of the DLC-ac4C model. It is worth noting that the ablation experiments of the model only change the corresponding module, other conditions remain the same. The model's performance is then evaluated to yield the results of a tenfold cross-validation of the model with independent testing. Finally, comparison with existing predictors. The results show that DLC-ac4C shows superior performance in all categories.

### 3.1 Contrasting Various Feature Extraction Techniques

To determine the most suitable encoding method for the DLC-ac4C model, we compared the performance of different encoding methods, including C2 encoding, NCP, and ND encoding (NCP+ND) and their hybrid encoding (C2+NCP+ND). In addition to this, to highlight the advantages of C2 encoding, we also compare C2+NCP+ND encoding with One-hot+NCP+ND encoding. Tables **4** and **5** lists the experimental results on the tenfold cross-validation and independent test datasets for the training dataset when each of the four different encoding methods is fed into the DLC-ac4C network framework, with the best results listed in bold.

From the experimental results in Tables **4** and **5**, it is easy to see that the evaluation indexes of the combined coding methods as sequence feature extraction are all higher than the results of using only one encoding method. In addition, on the tenfold cross-validation of the training dataset, the results of the two encoding combinations are compared, and the C2+NCP+ND coding method is higher than the one-hot+NCP+ND coding in all metrics except the lower Sn value. On the independent test set, C2+NCP+ND encoding had significantly higher Sp, Acc, and MCC values than One-hot+NCP+ND encoding. Therefore, it is reasonable to assume that combined coding is more effective than using one coding method alone, and in combined coding, C2 coding is tighter and can extract sequence features more efficiently than the sparseness of features extracted by One-hot encoding. In the end, we chose the C2+NCP+ND combined encoding method as the feature input to the model.

### 3.2 Comparison of Different Number of Dense Blocks

Since the amount of dense blocks in DenseNet is also an essential part of the model's performance, the parameters of DenseNet are optimized to improve the predictive performance of the model. In this section, we compare the model's performance by setting different numbers of dense blocks using Acc and MCC metrics. In Fig. (**8**) we compare Acc and MCC metrics for different numbers of dense blocks. It can be visually seen that when four dense blocks are stacked together, the model can achieve the highest performance, with both Acc and MCC being the highest. As the number of dense blocks increases, Acc and MCC values are likely to become higher, but considering that the higher the number of dense fast, the larger the maximum feature map scale will be when the model is running, which will easily drain the memory server. Therefore, in this work, four dense blocks were selected to construct the DenseNet.

### 3.3 Ablation Experiment for Model Architecture

Ablation tests were conducted to establish which of the different combinations of the four modules would be most suitable as a network framework for the model. The outcomes of the tenfold cross-validation are demonstrated in Tables **6** and **7** provides the outcomes of the independent tests. Tables **6** and **7** give a comparison of the performance of seven different combinations, where the results of the optimal combination are shown in bold. If a tick is marked in the corresponding row for each network method, it means that the method was selected for this experiment; if not, it means that the method was not selected.

As can be seen from Tables **6** and **7**, the results of the combination of the four modules are better than the other combination methods in Acc, MCC, and AUC, except Sn and Sp, which are slightly lower than the other combination methods. This is enough to show that each module plays an important role in the DLC-ac4C model, and the combination can be more effective in extracting the advanced features of the sequence. Therefore, we finally selected a combination of four modules as the network framework for the model DLC-ac4C.

### 3.4 Performance of DLC-ac4C on the Training Dataset

To better analyze DLC-ac4C's performance, we performed a 10-fold cross-validation on the training dataset. The ROC curve for the DLC-ac4C model on the training dataset for ten-fold cross-validation is plotted in Fig. (**9**), with a mean AUC of 0.8774. It can be clearly seen that the ten ROC curves are very stable, with a small overall difference and relatively small fluctuations, effectively avoiding the model's overfitting problem, and indicating that our proposed DLC-ac4C model has good stability.

### 3.5 Comparison with Different Machine Learning Algorithms

To make a comparison between deep learning and traditional machine learning on the ac4C site classification problem, we compared DLC-ac4C with other traditional machine learning algorithms, including Logistic Regression (LR), K-Nearest Neighbor (KNN), Random Forest (RF), AdaBoost (AB), Gaussian Naïve Bayes (NB), Support Vector Machine (SVM) and Gradient Boosting Decision Tree (GBDT). We use bar charts to represent the results of performance comparisons on independent test sets. As observed in Fig. (**10**) , it is apparent that the DLC-ac4C model exhibits the highest values for Sn, Acc, MCC, and AUC, as compared to the other seven machine learning methods. These results suggest that the DLC-ac4C model outperforms the others in predicting the ac4C site, indicating its suitability for ac4C site identification.

### 3.6 Comparison with Existing Predictors

Considering the availability and comparative rigor of existing prediction methods and proving further the robustness and superiority of the proposed model in a fair and prudent manner, therefore only iRNA-ac4C, a prediction method with the same dataset as in this study, was selected for cross-validation comparison. Table **8** shows the tenfold cross-validation results for the DLC-ac4C and iRNA-ac4C models. At the same time, we compared the four existing techniques on the same independent test set, and Table **9** shows the performance comparison on the independent test set.

The tenfold cross-validation results in Table **8** show that the DLC-ac4C model has higher Sn, Acc, MCC, and AUC than iRNA-ac4C. Although the tenfold cross-validation results are largely consistent with those of iRNA-ac4C, the results of the independent test set are significantly improved. As can be seen from Table **9**, the three predictors PACES, XG-ac4C, and DeepAc4C have very low results in other metrics, although their Sp is high. While DLC-ac4C increased Sn by 9.53%, Acc by 3.16%, MCC by 6.38%, and AUC by 2.42% compared to iRNA-ac4C, Sp decreased slightly. The radar plot in Fig. (**11**) for a visual comparison of the two predictors is available. This indicates that the DLC-ac4C model proposed in this study has a strong generalization ability and a strong predictive power to accurately identify potential ac4C sites.

## CONCLUSION

In this study, we built an integrated deep learning model called DLC-ac4C to predict ac4C sites in human mRNAs, which not only provides researchers with a reliable prediction tool and enriches research in the field of ac4C sites, but also contributes to the study of human beings with respect to various diseases.

Compared with other prediction models, the advantages of DLC-ac4C are shown in the following: First, we compare the coding methods of One-hot and C2 encoding respectively with the combination of NCP+ND and find that more effective prediction performance can be obtained by using the hybrid coding method of C2+NCP+ND to extract the original features of the sequences. Second, we constructed a network framework based on DenseNet and Bi-LSTM methods and embedded a channel attention module to extract high-level sequence features. Finally, we adopt the isomorphic integration strategy to improve the stability of the model. Experimental results show that the DLC-ac4C model proposed in this study has better prediction and generalization capabilities compared to existing models.

Although DLC-ac4C shows strong robustness in predicting ac4C sites, there are still some limitations. Firstly, the commonly used cross-entropy loss function was fixedly selected in this study, and there was no in-depth exploration from this perspective. Second, the dataset utilized in this study is relatively small and fails to account for the extensive data requirements of deep learning. Third, the CNN-based structure has the potential to lose the spatial relationship of the learned features.

In future research work, we can make the following extensions. First, the combined loss function [68] can be integrated into the model to improve prediction performance. Second, different data enhancement techniques [69-71] used in deep network models can be referenced and tried to be used to study ac4C site prediction. Third, a comparison can be made with capsule networks [72-74] that have the ability to preserve spatial relationships of the features studied. Additionally, all datasets and source code for the DLC-ac4C model are freely available at https://github.com/lencary/DLC-ac4C.

## Figures and Tables

**Fig. (1):**
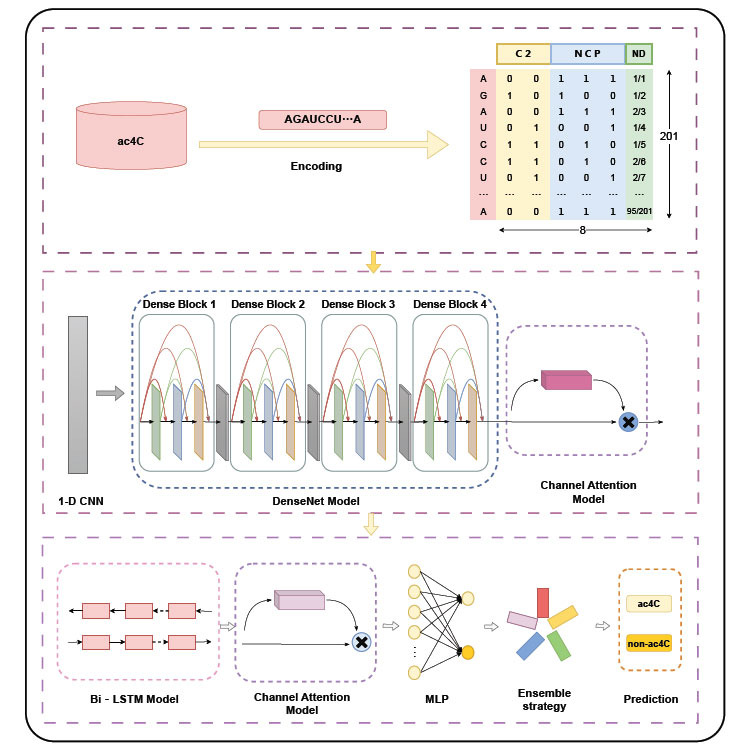
Overall flowchart of DLC-ac4C.

**Fig. (2):**
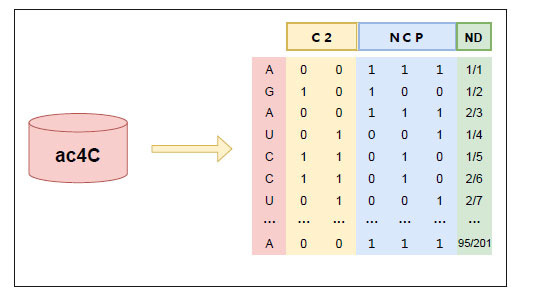
C2, NCP and ND encoding.

**Fig. (3):**
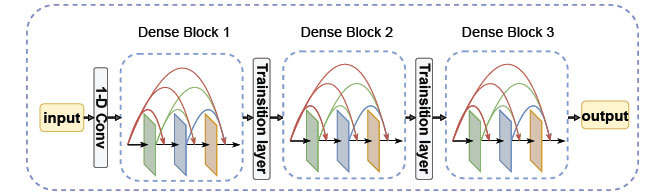
Structure of DenseNet.

**Fig. (4):**
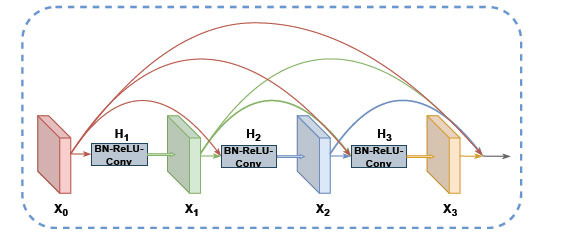
Structure of dense block.

**Fig. (5):**
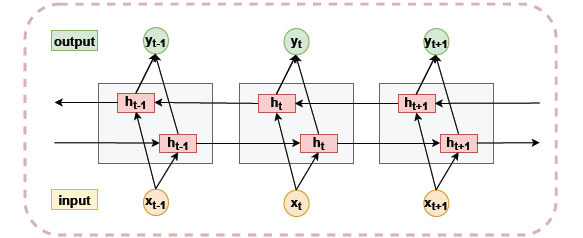
Structure of Bi-LSTM.

**Fig. (6):**
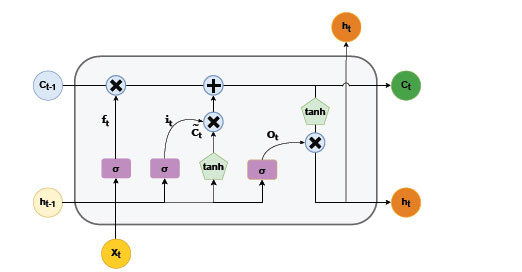
The schematic diagram of the LSTM cell.

**Fig. (7):**
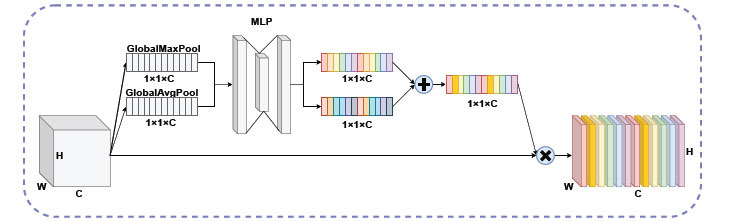
Channel attention mechanism.

**Fig. (8):**
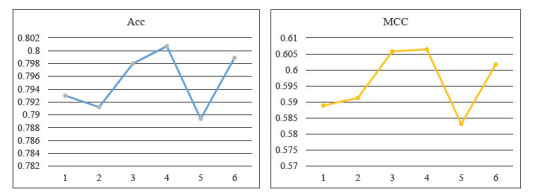
Compare ACC and MCC for different number of dense blocks.

**Fig. (9):**
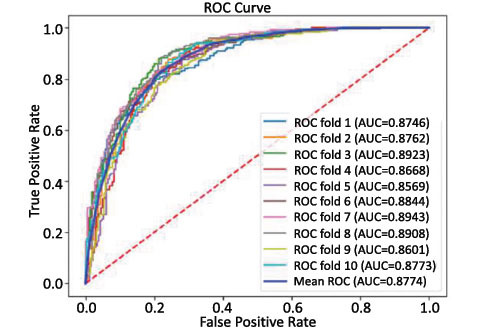
ROC curve for DLC-ac4C on the training dataset.

**Fig. (10):**
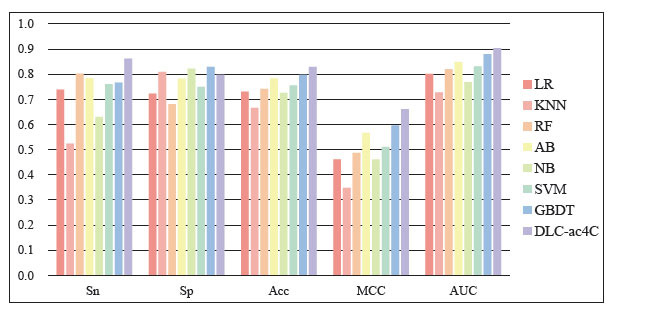
Performance comparison of different machine learning algorithms on independent test datasets.

**Fig. (11):**
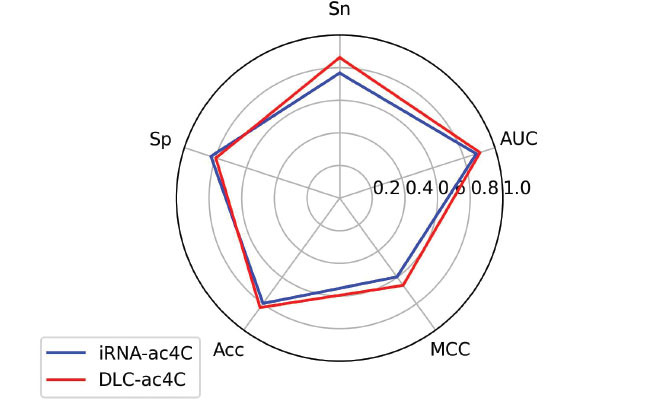
Comparison with iRNA-ac4C on the independent test dataset.

**Table 1:** Details of the benchmark dataset.

**Original**	**Training**	**Testing**
Positive	2206	552
Negative	2206	552
Total	4412	1104

**Table 2:** Nucleotide chemical property.

**Chemical Property**	**Class**	**Nucleotide**
Ring structure	Purine	A, G
	Pyrimidine	C, U
Functional group	Amino	A, C
	Keto	G, U
Hydrogen bond	Strong	C, G
	Weak	A, U

**Table 3:** Description of the hyperparameters for the DLC-ac4C model.

**Parameters**	**Number**
Dense block	4
Convolution layer number of a dense block	3
Convolution kernel size	96
Bi-LSTM layer neurons	240
Dropout ratio of DenseNet	0.5
First dense layer neurons	240
Dropout ratio 1	0.5
Second dense layer neurons	40
Dropout ratio 2	0.2
Last dense layer neurons	2

**Table 4:** Comparison of different encoding methods based on 10-fold cross-validation of the training dataset.

**Encoding**	**Sn**	**Sp**	**Acc**	**MCC**	**AUC**
C2	0.7615	0.5549	0.6581	0.3242	0.7183
NCP+ND	0.8354	0.7527	0.7921	0.5903	0.8737
C2+NCP+ND	0.8319	**0.7726**	**0.8007**	**0.6064**	**0.8774**
One-hot+NCP+ND	**0.8564**	0.7618	0.7937	0.5943	0.8746

**Table 5:** Comparison of different encoding methods based on independent test dataset.

**Encoding**	**Sn**	**Sp**	**Acc**	**MCC**	**AUC**
C2	0.7572	0.5435	0.6504	0.3078	0.7264
NCP+ND	**0.9257**	0.7011	0.8134	0.6433	0.9018
C2+NCP+ND	0.8623	**0.7971**	**0.8297**	**0.6608**	0.9036
One-hot+NCP+ND	0.8768	0.7772	0.8270	0.6573	**0.9042**

**Table 6:** Ablation experiments based on 10-fold cross-validation on the training dataset.

**DenseNet**	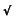	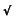	**-**	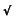	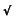	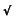	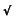
**Bi-LSTM**	-	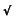	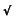	-	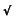	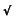	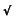
**Channel-attention**	-	-	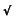	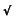	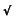	-	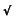
**Ensemble**	-	-	-	-	-	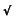	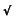
**Sn**	0.8415	0.8373	0.8015	0.7804	0.8206	**0.8550**	0.8319
**Sp**	0.7126	0.7433	**0.7931**	0.7926	0.7657	0.7344	0.7726
**Acc**	0.7735	0.7887	0.7960	0.7855	0.7923	0.7935	**0.8007**
**MCC**	0.5643	0.5875	0.5949	0.5806	0.5894	0.5940	**0.6064**
**AUC**	0.8649	0.8684	0.8739	0.8737	0.8704	0.8764	**0.8774**

**Table 7:** Ablation experiments based on independent test dataset.

**DenseNet**	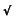	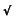	**-**	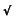	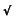	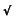	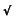
**Bi-LSTM**	-	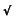	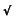	-	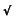	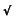	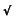
**Channel-attention**	-	-	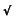	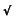	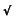	-	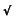
**Ensemble**	-	-	-	-	-	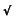	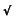
**Sn**	0.7011	0.8207	0.8370	0.6775	0.8641	**0.8659**	0.8623
**Sp**	**0.8804**	0.8243	0.7953	0.9022	0.8025	0.7862	0.7971
**Acc**	0.7908	0.8225	0.8161	0.7899	0.8233	0.8261	**0.8297**
**MCC**	0.5911	0.6449	0.6328	0.5949	0.6579	0.6543	**0.6608**
**AUC**	0.8950	0.8979	0.8935	0.9002	0.9015	0.9028	**0.9036**

**Table 8:** 10-fold cross-validation performance of DLC-ac4C and other predictors.

**Predictor**	**Sn**	**Sp**	**Acc**	**MCC**	**AUC**
iRNA-ac4c	0.7702	0.8301	0.8003	0.6010	0.8750
DLC-ac4C	**0.8319**	0.7726	**0.8007**	**0.6064**	**0.8774**

**Table 9:** Independent test dataset performance of DLC-ac4C and other predictors.

**Predictor**	**Sn**	**Sp**	**Acc**	**MCC**	**AUC**
PACES	0.0598	**1**	0.5299	0.1760	\
XG-ac4C	0.3587	0.8243	0.5915	0.2070	\
DeepAc4C	0.1007	0.9710	0.5362	0.1470	0.8030
iRNA-ac4c*	0.7670	0.8291	0.7981	0.5970	0.8800
DLC-ac4C	**0.8623**	0.7971	**0.8297**	**0.6608**	**0.9042**

**Note:** “The conclusions were from the previous study, as stated by the asterisk (*) [23]”

## Data Availability

The dataset and source code used in this study can be easily derived from https://github.com/lencary/DLC-ac4C.

## LIST OF ABBREVIATIONS

1-D CNN: One-dimensional Convolutional Neural Network
AB: AdaBoost
ac4C: N4 acetylcytidine
Acc: Accuracy
acRIP: RNA Acetylated RNA Immunoprecipitation
AUC: Area Under the Curve
Bi-LSTM: bi-directional Long Short-term Memory Networks
DenseNet: Densely Connected Convolutional Networks
f5C: 5-formylcytosine
GBDT: Gradient Boosting Decision Tree
hm5C: 5-hydroxymethylcytosine
KNN: K-Nearest Neighbor
LR: Logistic Regression
m7G: 7-methylguanosine
MCC: Mathews Correlation Coefficient
NAT10: N-acetyltransferase 10
NB: Gaussian Naïve Bayes
NCP: Nucleotide Chemical Property
ND: Nucleotide Density
ResNet: Residual Network Structure
RF: Random Forest
ROC: Receiver Operating Characteristic
Sn: Sensitivity
Sp: Specificity
SVM: Support Vector Machine
